# The Impact of Aging on Regulatory T-Cells

**DOI:** 10.3389/fimmu.2013.00231

**Published:** 2013-08-06

**Authors:** Johannes Fessler, Anja Ficjan, Christina Duftner, Christian Dejaco

**Affiliations:** ^1^Department of Rheumatology and Immunology, Medical University Graz, Graz, Austria; ^2^Department of Internal Medicine, General Hospital Kufstein, Kufstein, Austria

**Keywords:** FOXP3, regulatory T-lymphocyte, aging, cellular senescence, thymus, suppressor cells

## Abstract

Age-related deviations of the immune system contribute to a higher likelihood of infections, cancer, and autoimmunity in the elderly. Senescence of T-lymphocytes is characterized by phenotypical and functional changes including the loss of characteristic T-cell surface markers, while an increase of stimulatory receptors, cytotoxicity as well as resistance against apoptosis is observed. One of the key mediators of immune regulation are naturally occurring regulatory T-cells (T_regs_). T_regs_ express high levels of CD25 and the intracellular protein forkhead box P3; they exert their suppressive functions in contact-dependent as well as contact-independent manners. Quantitative and qualitative defects of T_regs_ were observed in patients with autoimmune diseases. Increased T_reg_ activity was shown to suppress anti-tumor and anti-infection immunity. The effect of aging on T_regs_, and the possible contribution of age-related changes of the T_reg_ pool to the pathophysiology of diseases in the elderly are still poorly understood. T_reg_ homeostasis depends on an intact thymic function and current data suggest that conversion of non-regulatory T-cells into T_regs_ as well as peripheral expansion of existing T_regs_ compensates for thymic involution after puberty to maintain constant T_reg_ numbers. In the conventional T-cell subset, peripheral proliferation of T-cells is associated with replicative senescence leading to phenotypical and functional changes. For T_regs_, different developmental stages were also described; however, replicative senescence of T_regs_ has not been observed yet.

## Introduction

The immune system combats against infectious agents and depletes damaged or transformed cells, whereas intact self-components are usually ignored. Nevertheless, clinical manifestations of autoimmunity occur in at least 5% of the general population. The exact causes of autoimmune diseases are elusive; however, genetic and environmental risk factors as well as an insufficient elimination of cells bearing autoreactive T-cell receptors (TCRs) in the thymus contribute to the evolvement of disease (1, 2). To prevent autoimmunity, tolerance mechanisms including clonal deletion, induction of apoptosis, or anergy of self-reactive T-cells are essential. In addition, regulatory T-cells (T_regs_) were identified as sentinels of the immune response keeping aberrant/exaggerated immune reactions in balance. Several distinct T-cell subsets with regulatory function have been identified so far including natural T_regs_, adaptive or induced T_regs_ (iT_reg_), type 1 regulatory T-cells (Tr1), T helper 3 cells (Th3), double-negative (dn) T-cells, γδ T-cells, and iNKT cells. In a number of autoimmune diseases a diminished prevalence and/or impaired function of T_regs_ were observed (3). As several autoimmune disorders (such as rheumatoid arthritis or vasculitis) occur more frequently in the elderly, the question arises whether aging is linked to quantitative and/or qualitative defects of the T_reg_ pool (4–6).

In this review we summarize current data about the effects of aging on T_regs_ and highlight the possible mechanisms leading to senescence of T_regs_.

## Characterization of T_regs_

### Definition and phenotype

Natural T_regs_ develop in the thymus through recognition of self-antigen presented by thymic epithelial or dendritic cells. For this process CD28 co-stimulation is required, whereas IL-2 and TGF-β are less important as indicated by knock-out mice models (7).

Today, there is still no consensus on the reliable identification of T_regs_ by flow cytometry. A variety of cell surface molecules have been proposed as specific T_regs_ markers such as glucocorticoid-induced tumor necrosis factor receptor (GITR), cytotoxic T-lymphocyte associated antigen-4 (CTLA-4), the co-receptors Neuropilin-1 and PD-1, the adhesion molecule CD62L, major histocompatibility complex (MHC) class II DR, or CD45 isoforms. The type I cytokine receptor CD127 is a negative marker of T_regs_ and the absence of this molecule is frequently used for T_reg_ identification (8).

The forkhead transcription factor FoxP3 was proposed as the most specific marker of T_regs_ as FoxP3 expression is essential for T_reg_ development and function (9): T_regs_ were unable to develop in a mouse receiving FoxP3-deficient progenitor cells from another animal (10) and retroviral expression of FoxP3 in human and murine T-cells enabled the conversion of non-regulatory naïve T-cells into a T_reg_-like phenotype with suppressive activity and surface expression of CD25 (9). A mutation of the *FoxP3* gene in humans results in the fatal autoimmune syndrome IPEX (immune dysregulation, polyendocrinopathy, X-linked) (11). For experimental studies, however, FoxP3 appears not to be an optimal T_reg_ marker because first, permeabilization of T-cells is necessary to stain FoxP3 and cells are thus not viable anymore and second, newer data indicate that human FoxP3 is up-regulated in activated T-cells without suppressive function as well (12).

The Ikaros family transcription factor Helios was proposed as an alternative indicator of human T_regs_ with a higher specificity compared to FoxP3. Recent data, however indicate that Helios is also up-regulated in activated non-regulatory T-cells (13). In summary, there is currently no specific marker of human T_regs_ available limiting the validity of studies investigating qualitative and/or quantitative changes of the T_reg_ pool.

### Mechanism of suppression

The mechanisms of T_reg_ mediated immunosuppression are still unclear. Most likely, T_regs_ have multiple functions with direct and indirect inhibitory effects on antigen-presenting cells (APCs) and T-cells such as the following (14, 15): (a) expression of the surface molecule CTLA-4 directly suppressing the activity of T-cells, (b) indirect inhibition of effector cells by the induction of anti-inflammatory biochemical pathways in APC, (c) direct or indirect killing of effector cells and APCs, and/or (d) production of immunoregulatory cytokines such as TGF-β and IL-10 (16).

Interestingly, a recent study reported that human T_regs_ are able to induce senescence of naïve and memory responder T-cells *in vitro* and *in vivo*. The resulting senescent T-cell subset had an altered phenotype and revealed potent suppressive functions. The mechanisms leading to senescence of non-regulatory T-cells were not completely understood; however, the phosphorylation of p38 and ERK1/2 signaling pathways inhibiting naïve T-cell growth and cell-cycle regulation appeared to play a role (17).

## The Effect of Aging on T_reg_ Prevalences and Function

A prevalence of approximately 0.6–15% out of the CD4^+^ T-cell pool has been reported for T_regs_ in healthy adults and mice (4, 18). The influence of aging on T_reg_ prevalence in humans has been rarely studied so far and available reports suggest only minor changes of the circulating T_reg_ pool through age (19). Higher proportions of T_regs_ were only found in cord blood samples suggesting a pivotal role of T_regs_ during homeostatic proliferation of naïve T-cells in the fetal life (20, 21). During the first 36 months of life T_reg_ levels decline rapidly (22) and remain relatively stable thereafter.

Mouse studies showed increased T_reg_ prevalences in lymphoid organs of aged compared to young animals, whereas frequencies in circulating blood and thymus were unchanged (23, 24). This finding led to the hypothesis that during aging T_regs_ accumulate in lymphoid tissues; hypothetically explaining the increased susceptibility to infections and reduced vaccine response in elderly animals. The accumulation of T_regs_ has further been observed in the skin of aged persons possibly resulting in a higher risk of skin cancer as T_regs_ reduce local anti-tumor immune responses (25–27).

In animals, T_reg_ function seems to decrease with advancing age. The transfer of CD25^+^ T_regs_ from aged mice into young animals for example resulted in a lower suppression of delayed type hypersensitivity responses compared to the infusion of young T_reg_ cells (23). Another study found that CD4^+^CD25^high^ T_regs_ from aged animals less efficiently inhibited the proinflammatory activity of IL-17^+^ T-cells compared to T_regs_ from young mice (28). In human studies it was observed that T_regs_ from young and elderly individuals similarly inhibited the proliferation of responder cells whereas the production of the anti-inflammatory cytokine IL-10 was reduced in cells from the older group. The phenotype of T_regs_ including expression of CD25, FoxP3, IL-7Rα, or chemokine receptor expression, however, was unchanged (29). In conclusion T_regs_ from aged individuals are less efficient in preventing the occurrence of autoimmunity, while their number remains unaltered.

On the other hand, cancer and infections occur more commonly in the elderly suggesting increased T_reg_ responses (see also above) (29–31). One mouse study found an increase of T_reg_ prevalences in aged animals correlating with a defective tumor clearance. CD25-depletion restored the anti-cancer immune response (32). Similarly, CD25-depletion in aged mice reduced the lesion size in a *Leishmania major* infection model (24). Others reported that the depletion of T_regs_ with denileukin diftitox improved tumor-specific immunity only in young mice whereas tumor growth was unaffected in aged mice. This was explained by increased numbers of myeloid-derived suppressor cell (MDSC) in aged animals, and upon depletion of these cells tumor-specific immunity was restored (33).

In summary, current data on age-related changes of T_reg_ prevalences and function are conflicting and do not completely explain the simultaneously increased risk of autoimmunity (suggesting lower T_reg_ function), cancer, and infections (indicating increased T_reg_ responses) in the elderly. Apart from the difficulty of a reliable identification of T_regs_ the possible accumulation of T_regs_ in lymphoid organs and/or tissues during aging might lead to an underestimation of the total T_reg_ pool in current human studies. Future studies investigating tissue samples from immune-organs of elderly individuals would be desirable to better understand the role of T_regs_ in the pathogenesis of age-related diseases.

## T_reg_ Development and Homeostasis

Development of natural T_regs_ in the thymus depends on a positive selection process including high affinity interactions of the TCR to cortically expressed host antigens. Thymic stromal lymphopoietin activated CD11c-positive dendritic cells (34), co-stimulatory molecules including CD28, PD-1, CD40L (35) as well as the cytokine IL-2 were all shown to be crucial for thymic T_reg_ generation (36–38). Besides, the Nr4a nuclear receptors (involved in apoptosis, proliferation, DNA repair, inflammation, and others) were recently reported to contribute to T_reg_ development. Mice lacking these receptors in T-cells were unable to produce T_regs_ and died early from systemic autoimmunity (39).

During aging a progressive degeneration of the thymus occurs leading to a substantial loss of its capacity to generate and export new T-cells (40, 41). Throughout middle age thymic epithelial space and the functional unit of thymopoiesis (and thus the production of T-cells) decline by approximately 3% per year until the age of 45 when only an irrelevant level of functional thymic tissue remains. The total number of T-cells in the periphery nevertheless is unchanged and peripheral mechanisms of T-cell renewal have to compensate for progressive thymic failure (42–44).

**Figure 1:**
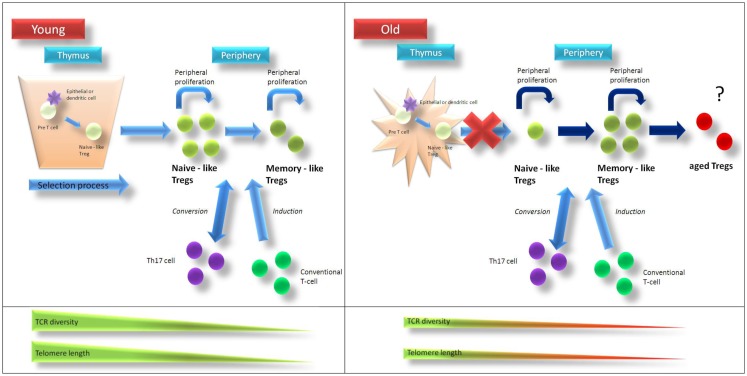
**Age-related changes of T_reg_ homeostasis**. In young individuals T_regs_ are generated in the thymus and are released as “naïve-like” T_regs_ into circulation. After antigen-contact, T_regs_ develop into a “memory-like” phenotype. T_reg_ homeostasis is supported by homeostatic proliferation of “naïve-like” and “memory-like” T_regs_ as well as conversion of non-regulatory T-cells into T_regs_. Telomere length and T-cell receptor diversity is higher in naïve-like compared to memory-like T_regs_. After puberty thymic function is progressively lost and in aged individuals homeostatic proliferation of existing T_regs_ as well as conversion of non-regulatory T-cells into T_regs_ compensate for thymic failure to maintain T_reg_ pool. Due to ongoing homeostatic replication telomere length and T-cell receptor diversity of T_regs_ from elderly people are contracted compared to those from young individuals. Recurrent stimulation of T_regs_ might then lead to a status of “terminal-differentiation” with altered phenotype and function. T_reg_ *regulatory T-cell*, TCR … *T-cell receptor*.

Parallel to the overall reduction of thymic T-cell output the production of thymically derived T_regs_ decreases with age (45). Alternative mechanisms such as increased surveillance of T_regs_ in the elderly (46) as well as peripheral T_reg_ generation may compensate for the loss of thymic function to maintain a sufficient T_reg_ pool (see Figure 1). Indeed, numerous studies indicate a possible conversion of non-regulatory CD4^+^CD25^−^ T-cells into T_regs_ *in vitro* and *in vivo* (47, 48). Moreover, mouse studies showed that peripheral self-antigen-driven proliferation of T_regs_ is a thymus-independent mechanism to maintain T_regs_ (49–51). The proportion of conventional T-cells differentiating into T_regs_ as well as the relative contribution of homeostatic T_reg_ proliferation to the overall T_reg_ pool in elderly individuals are unknown.

Peripheral mechanisms of T-cell renewal (particularly homeostatic expansion of existing T_regs_) are probably not infinite. Normally, T-cells proliferate beyond the seventh decade of life. Thereafter, telomere lengths are usually contracted to levels known as the “Hayflick limit”. At this stage, non-regulatory T-cells do not proliferate anymore and undergo phenotypical and functional changes such as down-regulation of CD28 and acquisition of cytotoxic potential (4, 52, 53). Due to the fact that T_regs_ display even shorter telomeres than non-regulatory T-cells, it is conceivable that peripherally proliferating T_regs_ reach the “Hayflick limit” even earlier (54). Impaired T_reg_ homeostasis may then result in immune dysfunction with increased risk of immune-mediated disorders.

In addition to the shortened telomere length, TCR diversity is also contracted to at least 100-fold in elderly individuals (55). This has been explained by the observation that homeostatic proliferation of T-cells is antigen dependent. Thus, T-cells with a high affinity TCR to self-antigens or antigens deriving from chronic virus infections have a survival advantage over other T-cells (42, 56). Given that similar mechanisms drive peripheral proliferation of non-regulatory T-cells and T_regs_, a reduction of T_reg_ TCR diversity (with a skew to certain antigens) can be expected in the elderly. Consequently, T_regs_ could mediate increased immunosuppression in response to specific self- (even if transformed) or viral antigens with increased incidence of malignancies and infections in the elderly. At the same time the reduced diversity of T_regs_ could result in decreased protection from autoimmunity (3).

## Development and Cellular Senescence of T_regs_

### From naïve to memory cell status

Similar to the developmental stages known for non-regulatory T-cells (development form CD45RA^+^ naïve to CD45RO^+^ memory and finally to CD28^−^ memory effector T-cells), different cellular subsets of T_regs_ were also observed. In humans, CD4^+^foxP3^+^ T_regs_ may have either a “naïve-like” phenotype characterized by the expression of CD25^+^CD45RA^+^ or a CD25^hi^CD45RO^+^ “memory-like” phenotype (54). In mice, naïve-like T_regs_ were characterized by the expression of CD25, CD62L, and CCR7 and by preferential homing to antigen-draining lymph nodes, where they were able to inhibit the induction of inflammation (10, 57, 58). Memory/effector-like T_regs_ (characterized by expression of CD29, CD44, ICOS, and LFA-1) migrated into non-lymphogenic tissues and sites of inflammation; a local down-regulation of immune reactions was shown (57, 58).

In humans, the highest prevalence of naïve-like T_regs_ were found in cord blood and it was assumed that these naïve-like T_regs_ are produced in the thymus (20, 59). The prevalence of memory-like T_regs_ increases rapidly during childhood and it was demonstrated that these memory-like T_regs_ have shorter telomeres and a lower content of TCR excision circles (Trecs) compared to naïve-like T_regs_ reflecting a longer replicative history (54). The mechanisms mediating the transition of a naïve-like T_reg_ into a memory-like phenotype still have to be explored; however, it is believed that antigen experienced dendritic cells migrating to secondary lymphoid tissues are involved. T_regs_ proliferate upon stimulation with autologous immature and mature dendritic cells (54, 60). A low surface expression of CD45RB on memory-like T_regs_ further supports the hypothesis of an antigen-driven development of naïve-like T_regs_. CD45RB is normally down-regulated after repeated antigen-contact (61).

Human adult peripheral blood usually contains both, naïve-like and memory-like T_regs_. Parallel to the reduction of total naïve T-cells, the quantity of naïve-like T_regs_ declines with age whereas the prevalence of memory-like T_regs_ increases (29, 62). The total pool of circulating T_regs_; however, remains unchanged as mentioned above (19). As naïve-like T_regs_ exhibit a higher proliferative potential *in vitro* compared to memory-like T_regs_ it can be expected that the capacity of the immune system to downregulate abnormal immune responses declines with age (54).

### End-differentiated T_regs_ and aspects of T_reg_ senescence

Replicative senescence of T-cells is a prominent feature of aging resulting from homeostatic proliferation and repetitive antigen exposure (63). The most important phenotypic feature of senescent T-cells is the loss of the type I transmembrane protein CD28, a major co-stimulatory molecule (64). From the functional perspective, non-regulatory CD28^−^ T-cells produce large amounts of interferon γ, perforin, and granzyme B, providing them with the ability to lyse target cells (65). Another feature of CD28^−^ T-cells is their longevity and persistence that can be explained by defects in the apoptotic pathway with upregulation of bcl-2 and Fas-associated death domain like IL-2-converting enzyme-like inhibitory protein (FLIP) (66, 67). Terminally differentiated T-cells also acquire new stimulatory receptors including killer cell immunoglobulin-like receptors (KIRs) and Toll-like receptors (TLRs) (68, 69). Thus, activation of CD4^+^CD28^−^ T-cells no longer depends on professional antigen-presenting cells, rather it is promoted by stress molecules as well as bacterial and/or viral products (65). The frequency of terminally differentiated CD4^+^CD28^−^ T-cells is increased in old individuals as well as in younger patients with autoimmune diseases such as rheumatoid arthritis or spondyloarthritis (70). Given that T_regs_ proliferate in the periphery to maintain the total T_reg_ pool after thymic failure it is plausible to hypothesize that T_regs_ may undergo terminal-differentiation as well.

Interestingly, a proportion of T_regs_ from aged mice showed decreased expression of CD25 (46, 71). These CD25^low^ T_regs_ occurred predominantly in the spleen (24) but had comparable functional properties to CD25^+^ T_regs_. A similar CD4^+^CD25^−^foxP3^+^ T_reg_ population has been observed in SLE patients. SLE patients are known to have a prematurely aged immune system (72) with accumulation of CD28^−^T-cells. A detailed characterization of CD4^+^CD25^−^FoxP3^+^ T_regs_ regarding the expression of naïve/memory T-cell markers or determination of telomere lengths was unfortunately not performed. Further evidence for the occurrence of T_reg_ senescence was found in a study on healthy aged individuals reporting the occurrence of a CD8^+^CD25^+^ T_reg_ population lacking CD28 expression. These regulatory cells shared phenotypic and functional features with CD4^+^ T_regs_ from the same population (73). The occurrence and possible characteristics of terminally differentiated CD4^+^ T_regs_ is an interesting issue that has to be investigated by future studies.

## Conclusion

Accumulating evidence suggests age-associated changes of T_reg_ prevalence and/or T_reg_ function. Due to involution of thymus after puberty peripheral mechanisms including homeostatic proliferation of T_regs_ or conversion of non-regulatory T-cells into T_regs_ compensate for the decreasing generation of new T_reg_ cells. However, these peripheral mechanisms are limited; this leads to altered composition of the T_reg_ pool. Age-related changes of T_regs_ are suspected to increase the risk of autoimmunity, cancer, and infections in the elderly; however, the exact mechanisms are still poorly understood. Current studies are limited by the difficult identification of human T_regs_ and the uncertainty whether circulating T_regs_ reflect the total T_reg_ pool or a cellular subset only. Future studies are required to investigate cellular senescence of T_regs_ and possible therapeutic approaches targeting T_regs_ in aged individuals.

## Conflict of Interest Statement

The authors declare that the research was conducted in the absence of any commercial or financial relationships that could be construed as a potential conflict of interest.
